# Clinical, biochemical, and pathophysiological analysis of *SLC34A1* mutations

**DOI:** 10.14814/phy2.13715

**Published:** 2018-06-19

**Authors:** Amy Fearn, Benjamin Allison, Sarah J. Rice, Noel Edwards, Jan Halbritter, Soline Bourgeois, Eva M. Pastor‐Arroyo, Friedhelm Hildebrandt, Velibor Tasic, Carsten A. Wagner, Nati Hernando, John A. Sayer, Andreas Werner

**Affiliations:** ^1^ Institute for Cell and Molecular Biosciences Medical School Newcastle University Newcastle United Kingdom; ^2^ Institute of Genetic Medicine Newcastle University Newcastle United Kingdom; ^3^ Division of Nephrology Department of Internal Medicine University Clinic Leipzig Leipzig Germany; ^4^ Institute of Physiology University of Zurich Zurich Switzerland; ^5^ Department of Medicine Boston Children's Hospital Harvard Medical School Boston Massachusetts; ^6^ Medical Faculty Skopje University Children's Hospital Skopje Macedonia; ^7^ Renal Services Newcastle Upon Tyne NHS Foundation Trust Newcastle United Kingdom

**Keywords:** Epithelial cell, Fanconi syndrome, metabolic acidosis, nephrolithiasis, phosphate, SLC34A1

## Abstract

Mutations in *SLC34A1*, encoding the proximal tubular sodium–phosphate transporter NaPi‐IIa, may cause a range of clinical phenotypes including infantile hypercalcemia, a proximal renal Fanconi syndrome, which are typically autosomal recessive, and hypophosphatemic nephrolithiasis, which may be an autosomal dominant trait. Here, we report two patients with mixed clinical phenotypes, both with metabolic acidosis, hyperphosphaturia, and renal stones. Patient A had a single heterozygous pathogenic missense mutation (p.I456N) in *SLC34A1*, consistent with the autosomal dominant pattern of renal stone disease in this family. Patient B, with an autosomal recessive pattern of disease, was compound heterozygous for *SLC34A1* variants; a missense variant (p.R512C) together with a relatively common in‐frame deletion p.V91A97del7 (91del7). *Xenopus* oocyte and renal (HKC‐8) cell line transfection studies of the variants revealed limited cell surface localization, consistent with trafficking defects. Co‐expression of wild‐type and I456N and 91del7 appeared to cause intracellular retention in HKC‐8, whereas the R512C mutant had a less dominant effect. Expression in *Xenopus* oocytes failed to demonstrate a significant dominant negative effect for I456N and R512C; however, a negative impact of 91del7 on [^32^P]phosphate transport was found. In conclusion, we have investigated pathogenic alleles of *SLC34A1* which contribute to both autosomal dominant and autosomal recessive renal stone disease.

## Introduction

The maintenance of plasma phosphate is an important physiological function which requires an interplay between intestinal absorption of phosphate, storage in the bones, and excretion by the kidneys (Forster et al. 2011). Only a small percentage of total body of phosphate is present within the plasma; however, this is regulated tightly by hormones including parathyroid hormone (PTH), calcitriol, fibroblast growth factor 23 (iFGF‐23), and its cofactor klotho. Both iFGF‐23 and klotho promote renal excretion of phosphate by regulation of proximal tubular phosphate transport proteins (Kuro and Moe 2017). Indeed, a set of renal proximal tubular phosphate transporters including the sodium–phosphate cotransporters encoded by *SLC34A1* and *SLC34A3* allow for reabsorption of 70–80% of filtered phosphate (Table 1). The renal‐specific sodium–phosphate cotransporter encoded by *SLC34A1* is termed NaPi‐IIa and is located at the apical brush border of the proximal tubule where it allows reabsorption of filtered sodium and phosphate (Sayer 2017).

**Table 1:** Summary of sodium–phosphate cotransporters type II in man

Gene	Protein	Stoichiometry	Phylogeny	Tissue distribution	Linked disorder
*SLC34A1*	NaPi‐IIa	3 Na^+^/1 P_i_ electrogenic	Mammals, vertebrates	Renal proximal tubules	Nephrocalcinosis Infantile hypercalcemia Fanconi renotubular syndrome
*SLC34A2*	NaPi‐IIb	3 Na^+^/1 P_i_ electrogenic	Mammals, vertebrates	Small intestine, lungs, testis, mammary gland	Pulmonary alveolar microlithiasis
*SLC34A3*	NaPi‐IIc	2 Na^+^/1 P_i_ electroneutral	Mammals	Renal proximal tubules	Hypophosphatemic rickets with hypercalciuria

Mutations in *SLC34A1* are known to give several different clinical disease phenotypes. These include an autosomal recessive form of infantile hypercalcemia, where biallelic mutations result in loss of function of NaPi‐IIa leading to phosphate depletion. This gives rise to a decrease of iFGF‐23 levels and results in an unrestricted activation of 1,25‐(OH)_2_D_3_ producing a phenotype of hypercalcemia, hypercalciuria, and nephrocalcinosis (Schlingmann et al. 2016; Wagner et al. 2017). Biallelic mutations in *SLC34A1* may also cause an autosomal recessive Fanconi‐like renotubular syndrome. A homozygous in‐frame 21‐bp insertion duplication mutation (p.I154_V160dup) was reported in a consanguineous family where two affected siblings had a hypophosphatemic rickets phenotype together with some signs of generalized proximal tubulopathy (Magen et al. 2010).

In addition to these recessively inherited disease phenotypes, autosomal dominant forms of disease have been seen with single heterozygous changes in *SLC34A1*, notably hypophosphatemic nephrolithiasis/osteoporosis‐1. Two unrelated patients with biochemical hypophosphatemia and reduced renal phosphate reabsorption have been reported (Prie et al. 2002). This included a man with recurrent renal stones and a woman with evidence of bone demineralization.

Murine models of *SLC34A1* have been described. Homozygous *Slc34a1* ^*−/−*^ knockout mice demonstrated increased urinary phosphate levels, hypophosphatemia, and elevated 1,25‐(OH)_2_D_3_ levels. Young animals exhibited bony defects including poorly developed trabecular bones and retarded secondary ossification which improved with age (Beck et al. 1998). Heterozygous mice were healthy but did have a mild biochemical phenotype. Serum phosphate was normal, but the mice had evidence of phosphaturia and had raised serum 1,25‐(OH)_2_D_3_ levels (Beck et al. 1998).

Here, we present the clinical, genetic, and biochemical data of two unrelated patients, in whom mutations in *SLC34A1* have resulted in different but overlapping phenotypes. We use in vitro modeling of the mutations to determine their pathogenicity and contribution to loss of renal phosphate handling.

## Methods

### Clinical, biochemical, and genetic analysis

Patients gave informed written consent to these studies. Clinical data were reviewed. DNA was obtained from patients and relatives where available. The study was approved by the Newcastle upon Tyne Research Ethics Committee. DNA was extracted from whole‐blood samples. Next‐generation sequencing of a renal stone panel was performed as previously described (Halbritter et al. 2015), and variants/segregation was confirmed by Sanger sequencing. In silico tools and database searches were used to determine pathogenicity of variants and allele frequency. Plasma levels of iFGF23 and 1,25(OH)2 vitamin D_3_ were determined with an ELISA and radioimmunoassay kits, respectively (Immunotopics International, USA; Immunodiagnostic System, Germany).

### In silico modeling of *SLC34A1* mutations

Mutations in *SLC34A1* were modeled using a previously reported NaPi‐IIa homology model (Fenollar‐Ferrer et al. 2014). Figures were prepared using PyMOL (http://www.pymol.org/).

### Molecular biology and expression studies

Site‐directed mutagenesis was performed using the Quickchange Lightning Kit (Agilent) and mutations were confirmed by sequencing. The open reading frames for green fluorescent protein (GFP) and red fluorescent protein (RFP) were introduced to the *SLC34A1* sequence by overlapping PCR either at the 3′ or 5′ end. A plasmid for transfection control (GFP alone) was generated by introducing a frame shift to the *SLC34A1* open reading frame immediately after the start codon. For oocyte injections, plasmids were linearized using XbaI and in vitro transcribed using the T7 mMessageMachine kit (ThermoFisher).

*Xenopus* oocytes were purchased from Ecocyte (Germany) and incubated in Barth's solution. Routinely, 10 ng of in vitro synthesized RNA was injected, and [^32^P]phosphate flux measurements were performed after 3–5 days (Markovich 2008).

HKC‐8 cells were cultured as previously described (Hernando et al. 2000). Cells were transfected with N‐terminally linked GFP/RFP‐SLC34A1 constructs using Lipofectamine 2000 (ThermoFisher). Cell nuclei were stained with DAPI and plasma membranes with either rhodamine‐coupled phalloidin or wheat germ agglutinin (WGA) (Vector Laboratories) prior to imaging by confocal microscopy (Nikon A1). Images were deconvolved (Huygens Professional) and analyzed using NIS‐Elements software (Nikon).

### Preparation and analysis of urinary exosomes

Urinary exosomes were collected from 15‐mL urine by serial centrifugation as previously reported (Pathare et al. 2018). The final pellet was resuspended in Laemmli buffer (0.6% *w*/*v* SDS, 3% *v*/*v* glycerol, 18 mmol/L Tris‐HCl pH 6.8, and 0.003% *w*/*v* bromophenol blue).

## Case Reports

Patient A from the United Kingdom was referred to the nephrology department at the age of 26 years for investigation of raised serum alkaline phosphatase and low serum phosphate. His medical history was noteworthy in that the patient had sensorineural deafness, epilepsy, learning difficulties, and renal impairment. There was an autosomal dominant family history of renal stones, with the patient's mother being affected with recurrent calculi, but no other clinical features. At the time of assessment, he was not taking any medication and blood pressure was normal (110/70 mmHg). Urine dipstick confirmed glycosuria (in the context of a normal blood glucose). Serum and urine biochemistry initially showed hypophosphatemia and renal phosphate wasting which then normalized in the context of progressive chronic kidney disease (CKD) (Table 2). In the presence of CKD stage 4 (eGFR 23 mL/min/1.73 m^2^), serum iFGF23 levels were raised and 1,25‐(OH)_2_D_3_ were reduced. Renal ultrasound scanning showed a single 7‐mm calculus in the lower pole of the left kidney and smaller calculi in the right kidney. A screen for urinary amino acids revealed a generalized aminoaciduria consistent with a proximal tubulopathy. Genetic investigations using next‐generation sequencing of a targeted panel of renal stone genes revealed a single heterozygous change in *SLC34A1* (c.1367T>A; p.I456N) which was confirmed by Sanger sequencing. There were no pathogenic variants in the related phosphate transporter *SLC34A3*. Segregation of the mutation from the parents was not possible due to unavailability of DNA samples; however, it is likely that the deafness, learning difficulties, and epilepsy seen in the proband but not the mother may be explained by another, as yet unidentified cause. The patient was treated with phosphate and low‐ dose vitamin D supplementation.

**Table 2:** Clinical, imaging, and biochemical features of patients

	Patient A (UK)	Patient B (Macedonia)
Age (years)	48	1
Renal imaging	Bilateral radiopaque calculi	Bilateral medullary nephrocalcinosis
Plasma phosphate (mmol/L)	0.7–1.2 (↓‐↔)	0.52 (↓)
Plasma bicarbonate (mmol/L)	17	20
Plasma calcium (mmol/L)	2.1 (↔)	2.1 (↔)
Plasma creatinine (*μ*mol/L)	265 (↑)	36(↔)
eGFR (mL/min/1.73 m^2^)	23 (CKD‐EPI)	66 (New Schwartz formula)
Total Vitamin D (nmol/L)	89	N/A
1,25‐(OH)_2_ D_3_ (pg/mL)	30 (↓)	24 (↓)
iFGF23 (ng/L)	380 (↑)	152 (↑)
Urine pH	7.0	5.5
Urine calcium/creatinine ratio (mmol/mmol creatinine)	0.315(↔)	0.42(↔)
Fractional excretion of phosphate (NR 10–20%)	79% (↑)	49% (↑)

CKD‐EPI, Chronic Kidney Disease Epidemiology Collaboration formula for estimating GFR; eGFR, estimated glomerular filtration rate; N/A, not available; NR, normal range.

**Figure 1:**
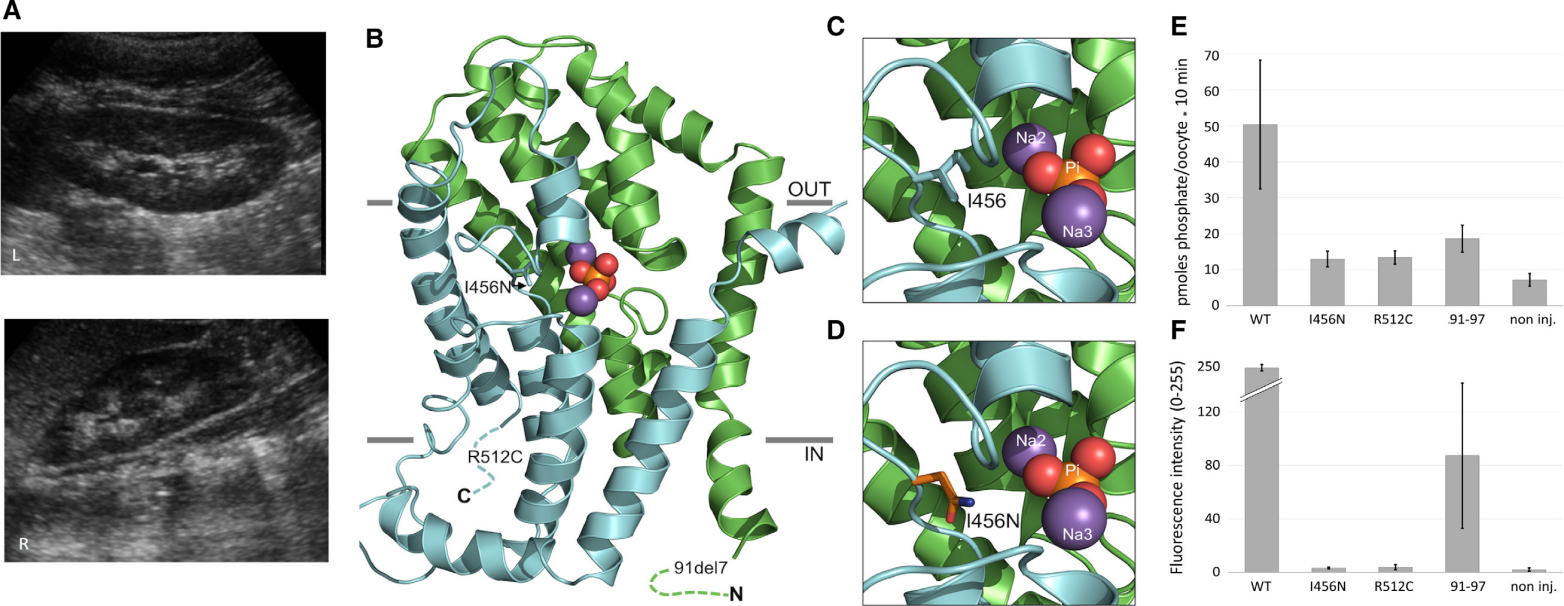
Clinical, structural, and functional characterization of *SLC34A1* mutations. (A) Medullary nephrocalcinosis seen in patient B. Renal ultrasound images of left and right kidneys are shown, demonstrating bilateral nephrocalcinosis. (B, C, D) Homology model of NaPi‐IIa (residues 97–502) with the two structural repeats (RU1 and RU2) highlighted in green and cyan, respectively. Bound phosphate (Pi) is shown as orange and red spheres and sodium ions as magenta spheres. Note that only approximate positions of the 91del7 and R512C mutants are indicated. The hydrophobic isoleucine at position 456 (I456) in RU2 of wild‐type NaPi‐IIa (C) is replaced in (D) by the polar asparagine residue (I456N). (E) *Xenopus* oocyte transport activity of wild‐type (WT) and mutated *SLC34A1* **‐** GFP‐coupled constructs. Transport activity was determined by [^32^P]phosphate flux measurements. WT significantly stimulates [^32^P]phosphate uptake compared with non‐injected oocytes (*P* < 0.01, ANOVA post hoc Tukey). The slightly increased transport rate with the mutants is not significant. (B) Surface expression of *SLC34A1*‐GFP constructs in *Xenopus* oocytes assessed by fluorescence microscopy. Fluorescence was quantified using image J, the average intensity of eight oocytes is shown (range 0–255 AU).

Patient B from Macedonia presented at 7 months of age with severe hypophosphatemia and renal phosphate wasting and a normal anion gap metabolic acidosis (Table 2). The child was initially acutely unwell with hypovolemia. Serum iFGF23 levels were raised and 1,25‐(OH)_2_D_3_ were reduced, which are not typical of NaPi‐IIa mutations and may reflect the clinical status of the patient. Serum iFGF23 levels are reported to rise rapidly in cases of sepsis and acute kidney injury. Renal ultrasound scanning revealed bilateral medullary nephrocalcinosis (Figure 1A) and metabolic acidosis (serum bicarbonate 16 mmol/L). Serum iFGF23 levels were raised and 1,25‐(OH)_2_D_3_ were reduced. There was no other family history of renal or stone disease. The patient was managed with bicarbonate and phosphate supplementation. Genetic investigations (as above) revealed biallelic mutations in *SLC34A1* [c.1534C>T; p.R512C and c.271_291del21; p.V91A97del7 (91del7)], which were confirmed by Sanger sequencing. The deletion segregated from the paternal allele while the missense change segregated from the mother.

### In silico assessment of *SLC34A1* variants

In silico analyses of the *SLC34A1* variants are summarized in Table 3. Variant c.1367T>A (I456N) has not been reported previously and has a very low allele frequency in the ExAC database. Variant c.1534C>T (R512C) is also rare, whereas variant c.271_291del21 (91del7) is more common. All mutations are predicted to be pathogenic (Table 3), with the position of the I456N mutation, close to the NaPi‐IIa substrate binding pocket (Figure 1B–D), consistent with a deleterious effect on transporter function. The NaP‐IIa homology model, covering residues 97–502 (Fenollar‐Ferrer et al. 2014), did not allow for insights as to the structure‐function effects of the 91del7 and R512C mutations. However, previous reports suggest that the 91del7 mutation affects protein trafficking (Lapointe et al. 2006; Schlingmann et al. 2016). R512 (and I456) are evolutionarily highly conserved, indicative of functional importance.

**Table 3:** In silico assessment of *SLC34A1* mutations

Nucleotide change (Ref sequence NM_003052)	Amino acid change	Amino acid conservation	MutationTaster	PolyPhen2	SIFT	ExAC frequency	References
c.1367T>A	I456N	To nematode^a^	Disease causing	Probably damaging	Deleterious	3 het alleles in 119,548 alleles	Novel
c.1534C>T	R512C	To nematode^a^	Disease causing	Possibly damaging	Deleterious	8 het alleles in 121,334 alleles	Halbritter et al. (2015)
c.271_291del21	91del7	n/a	Disease causing	n/a	n/a	17 hom alleles and 2148 het alleles in 121,274 alleles	Lapointe et al. (2006) and Schlingmann et al. (2016)

Het, heterozygous; hom, homozygous.

a GenBank accession KRZ90293.1

### Functional characterization of *SLC34A1* mutations

To determine the impact of the *SLC34A1* mutations on the function of NaPi‐IIa, fluorescently labeled transporters were expressed in *Xenopus laevis* oocytes and a renal epithelial (HKC‐8) cell line. NaPi‐IIa mutants I456N, R512C, and 91del7 exhibited significantly reduced [^32^P]phosphate transport compared with wild‐type NaPi‐IIa injected *Xenopus* oocytes (Figure 1E). As reported previously (Schlingmann et al. 2016), the 91del7 mutant showed some residual activity when compared with water‐injected controls (Figure 1E). Fluorescence microscopy confirmed membrane localization of wild‐type NaPi‐IIa, and, to a lesser extent, the 91del7 mutant (Figure 1F). However, the I456N and R512C mutants failed to reach the oocyte membrane.

**Figure 2:**
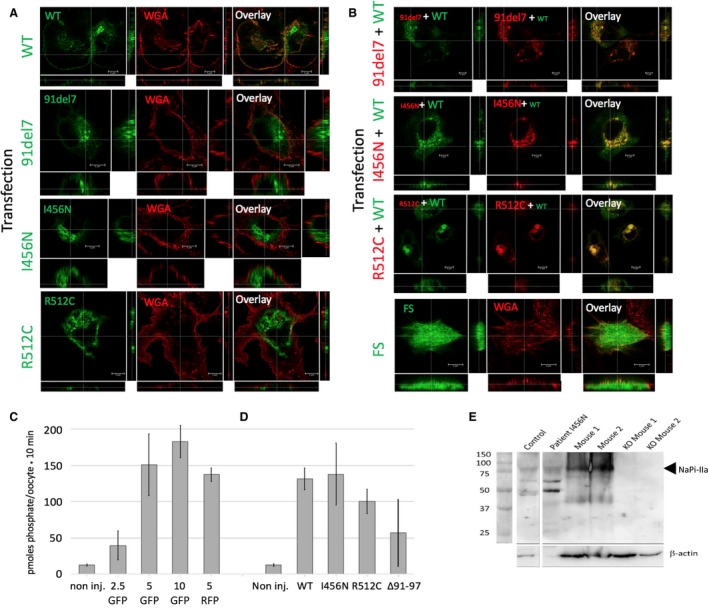
jeeFunctional assessment of *SLC34A1* mutations in vitro and phenotyping of urinary exosomes. (A–B) Assessment of transfected *SLC34A1* constructs in HKC‐8 cells. (A) Immunofluorescence images (subapical *xy* sections as well as *xz* and *yz* sections) of N‐terminal GFP transfected *SLC34A1* constructs (green) and wheat germ agglutinin lectin membrane staining (WGA, red). The wild‐type (WT) and mutant *SLC34A1* constructs (91del7, I456N, R512C) used are indicated. The white line indicates the cross‐section. Scale bar 5 *μ*m. (B) Co‐transfections of WT GFP‐*SLC34A1* construct (first column, specified at the left of each panel) and N‐terminal RFP‐*SLC34A1* mutant constructs (middle column) and overlay. Bottom row shows a GFP *SLC34A1* construct with a frame shift after the start codon (FS) as transfection control and WGA staining (red). Scale bar 5 *μ*m. (C–D) Co‐expression of WT and mutated *SLC34A1* in *Xenopus* oocytes. (C) Dose–response measurements. Amount (ng) of injected GFP or RFP‐tagged *SLC34A1* RNA are shown. (D) Co‐injection of 5 ng of WT with the same quantity of mutated *SLC34A1* RNA. (E) Western blot of exosomes isolated from human and mouse urine. Control used urine from an aged‐matched healthy human. Mouse 1 and 2 are healthy controls, KO are murine knockout (null allele) for NaPi‐IIa. Blots were probed with a polyclonal NaPi‐IIa‐specific antiserum that recognizes both human and mouse isoforms (75–80 kDa). Samples were loaded to match serum creatinine levels. Beta‐actin (42 kDa) was used as a loading control.

To investigate the reduced membrane expression of the missense variants I456N and R512C in a mammalian model system, N‐terminally tagged GFP and RFP constructs were expressed in HKC‐8 cells (Figure 2A and B). In HKC‐8 cells, wild‐type (WT) NaPi‐IIa showed predominantly apical localization with some residual punctate intracellular staining in the subapical region (Figure 2A). All the mutants showed predominantly intracellular staining and protein accumulation in perinuclear compartments, indicating defective protein folding and/or trafficking to the plasma membrane. A NaPi‐IIa construct (FS) with a premature termination codon showed a homogenous fluorescence without clusters, reminiscent of cytoplasmic GFP staining (Figure 2B, bottom).

To test potential interactions between mutated and wild‐type transporters, co‐expression experiments were performed. Co‐transfections of RFP‐tagged mutated *SLC34A1* constructs (91del7, I456N, and R512C) with GFP‐tagged wild‐type NaP‐IIa resulted in much of the protein being retained intracellularly in perinuclear, granular structures containing wild‐type and mutated transporters. Some of the wild‐type transporters fail to reach the periphery, suggesting a negative effect, of the mutated forms on wild‐type protein sorting (Figure 2B). The complex nature of the efficient membrane delivery was also confirmed when WT and mutated constructs were assessed in *Xenopus* oocytes. Increasing amounts of WT RNA (2.5–10 ng) concomitantly increased [^32^P]phosphate transport, and transport activity was not influenced by the C‐terminal GFP or RFP tag (Figure 2C). Co‐expression of 5 ng WT RNA with equal amounts of I456N or R512C mutant RNA failed to significantly reduce [^32^P]phosphate transport activity. However, co‐expression of WT and 91del7 RNAs caused some reduction in [^32^P]phosphate transport (Figure 2D). Finally, urine‐derived exosomes were obtained from patient A, and these confirmed the presence of NaPi‐IIa detected by Western blotting, consistent with a negative but not fully dominant effect of the heterozygous p.I456N missense variant (Figure 2E).

## Discussion

The continued investigation of potential pathogenic alleles in *SLC34A1*, which encodes the renal sodium–phosphate transporter NaPi‐IIa, is important in determining the role of NaPi‐IIa in proximal tubular function. Here, we demonstrate clinical phenotypes occurring in both dominant and recessive patterns. Autosomal dominant variants in *SLC34A1* are important to study, given that previous reports of phenotypes are rare and are limited to renal stone phenotypes and bone mineralization defects (Prie et al. 2002; Braun et al. 2016). There is a clear role for NaPi‐IIa in proximal renal tubular phosphate reabsorption and mutations in *SLC34A1* leading to renal stones (Sayer 2017). Previous studies of cohorts of pediatric stone formers have identified single heterozygous variants in genes, including *SLC34A1*, associated with autosomal dominant inheritance patterns (Braun et al. 2016). In this cohort, 5 of 143 individuals (3.5%) had heterozygous *SLC34A1* variants associated with nephrolithiasis. However, the possible dominant negative effects of a heterozygous mutation in *SLC34A1* on other proximal renal tubular transporters, producing a renal Fanconi syndrome, is harder to explain. Given the extra‐renal manifestations in patient A and the atypical clinical course, other underlying genetic changes may be implicated in the overall phenotype. The *SLC34A1* missense mutation I456N we report here is novel and our data point toward a trafficking defect, consistent with the putative proximity of the mutation to the functionally critical substrate‐binding domain and is consistent with the proximal tubulopathy phenotype we describe.

Both patients reported in this study exemplify the complex consequences of mutations in *SLC34A1* and the challenges characterizing them experimentally. Large‐scale genome sequencing projects, in combination with matching patient data, point to a significant role of the genetic background in mitigating phenotypic penetrance of deleterious *SLC34A1* mutations (Xue et al. 2012). Mutations in *SLC34A1* show more severe and early‐onset phenotypic consequences if both alleles are affected. Accordingly, patient B presented with severely disturbed phosphate balance before the age of 1 carrying two mutated *SLC34A1* alleles (91del7 and R512C). We found that both mutations significantly reduce phosphate transport in oocytes. A significant variability between cells suggests dosage effects and a strong influence of genetic/cellular background on the phenotype. Intercellular variability may also explain the discrepancy of our findings to Schlingmann et al. (2016) who analyzed the 91del7 mutation and found normal transport but impaired apical sorting in OK cells.

We tested whether dominant effects of single mutations could be recapitulated using *Xenopus* oocytes and a renal cell line. In oocytes, there was a slight but not significant decrease in transport upon co‐injection of WT and I456N/R512C RNA, whereas co‐expression of 91del7 with WT caused a reduction in WT‐induced transport. Moreover, all of the mutations appear to have a negative effect on the sorting of the wild‐type transporter. The fact that the 91del7 mutation is found in about 2% of the population (Wagner et al. 2017) may emphasize the importance of the cellular background on the penetrance of a particular mutant.

To conclude, we report two patients with mutations in *SLC34A1* with severely affected phosphate homeostasis. Characterization of the mutations confirms the deleterious effect on function and membrane delivery and points to a yet uncharacterized contribution of the genetic/cellular background to the penetrance of the phenotype.

## Conflict of Interest

The authors have no conflicts of interest to declare.
